# Prevalence of anemia and its associated factors among under-five age children in Shanan gibe hospital, Southwest Ethiopia

**DOI:** 10.1186/s12887-021-03011-5

**Published:** 2021-12-03

**Authors:** Destaw Kebede, Fantahun Getaneh, Kirubel Endalamaw, Tariku Belay, Abebe Fenta

**Affiliations:** 1 Department of Diagnostic Laboratory at Shegaw Motta General Hospital, East Gojjam, P.O. Box 50, Motta Town, Ethiopia; 2 Institute of Health Science, School of Medical Laboratory Science, Jimma University, Jimma Town, Ethiopia; 3 Department of Medical Laboratory Science, College of medicine and Health Science, Debre Markos University, Debre Markos Town, Ethiopia

**Keywords:** Anemia, Prevalence, Under five children, Associate factors, Southwest Ethiopia

## Abstract

**Background:**

Anemia is a major health problem in the worldwide. Because of health and socioeconomic problems, the prevalence of anemia is higher in developing countries. However, there was a limited finding in our study area. Therefore, the aim of this study was to determine the prevalence of anemia and its associated factors among under-five age children in Shanan Gibe Hospital (SGH), Southwest Ethiopia.

**Methods:**

Institution based cross sectional study was conducted at SGH, Ethiopia using consecutive convenient sampling technique during 1 January to 30 April, 2021. Data was collected by interviewing and capillary blood was taken from the fingertip for hemoglobin determination by using HaemoCue digital photometer. Additionally, stool sample was processed using wet mount and formal-ether concentration technique. Then after, the data were entered to Epidata version 3.1 and analysed with Statistical Package for the Social Sciences (SPSS) version 20. Factors associated with anaemia were assessed by bivariable and multivariable logistic regression model by considering *P* < 0.05 as statistical significance.

**Results:**

A total of 368 under five children were recruited to the study and the current prevalence of anemia was 48.9%. Of this anemia, 25.0% mild, 15.8% moderate and 8.2% were severely anemic. More ever, being rural resident (AOR = 6.11; 95% CI = 1.49–8.99, *P* = 0.002), family low income (AOR = 6.27, 95% CI = 1.35–11.43, *P* = 0.004), family size greater than five (AOR = 3.12; 95% CI =1.47–7.11, *P* = 0.002) and intestinal parasite infections such as *Enteameoba histolytica* (AOR =3.37; 95%CI = 2.16–11.31, P = 0.005), Hookworm (AOR = 6.09; 95%CI = 2.37–11.56, *P* = 0.001), and *Trichuris trichuria* (AOR = 2.79; 95%CI = 1.45–9.13, *P* = 0.002) (*P* < 0.05) were factors significantly associated with anemia among under five children.

**Conclusion:**

The current prevalence of anemia among under five age children is relatively high. On the other hand, the rural residence, large family size, low family income, infection with *Enteameoba histolytica*, hookworm and *Trichuris trichuria* were the identified factors associated with anemia among under five children. Therefore, there should be massive and routine deworming program in addition to imperative targeting anemia prevention, and nutritional supplementation to reduce the burden of anemia.

## Background

Anemia is a low number of red blood cells or a low hemoglobin or hematocrit in which the hemoglobin content of the blood is lower than normal range as a result of deficiency of one or more essential nutrients [1] or due to heavy blood loss, parasitic infections and congenital hemolytic diseases. Types of anemia includes iron deficiency anemia, sickle cell anemia, Vitamine deficiency anemia, Aplastic anemia, hemolytic anemia and anemia of inflammation [2] According to World Health Organization a child less than five years old is anemic if the blood hemoglobin is less than 110 g per liter (Hgb < 11.0 g/dl) [3].

Globally, approximately 1.62 billion people are affected by anemia [4] and approximately 36.4 to 61.9% of under five children in sub-Saharan Africa are also affected [5]. Similarly, it is considered to be a serious public health problem in Sub Saharan Africa in which, approximately 83.5 million children was affected and its prevalence of anemia was 67% [6]. The risk factors for anemia vary in different settings; they include intestinal worms, malaria, HIV infection, nutritional deficiencies, hematological malignancies and chronic diseases like sickle cell disease [6, 7].

Globally, anemia is a public health problem affecting people in both developed and developing countries with bad consequences of human health as well as social and economic development. It is also associated with increased morbidity and mortality [8]. Anemia affects all age group especially, under-five age children from low income families have a higher risk for developing anemia due to iron deficiency that occurs as a result of high demand for iron during the period of rapid growth [9].

In developing countries having low-and middle-income, the prevalence of anemia among 6–59 month age children was >20% based on latest demographic and heath survey (DHS) report rounds between 2005–2018, and it is classified as severe public health problem [10]. The problem is alarming in Sub-Saharan African Countries such as Kenya 48.9% [11]; Mali 55.8% [12] and Tanzania 79.6% [13]. Lack of awareness among the mothers about the problem coupled with their low educational status [14], poor nutritional practices and unhealthy food habits [15], low iron bioavailability of the diet [16], decreased physical activities [17], malaria and parasitic infestations are additional factors associated with lower hemoglobin (Hgb) level in children [18]. Factors including family size, low socio-economic status, illiteracy and ignorance are associated with anemia among under five children. Infection with Hook worm and intestinal helminthes causes gastro-intestinal blood loss resulting in depletion of iron stores and consequently also impaired erythropoietin [7]. This leads to mal-absorption and inhibition of appetite, there by worsening micronutrient deficiency and children anemia [19]. The consequence of anemia in under five age children include: decrease mental performance, low tolerance to infection, death from anemic heart failure [17, 19].

Although anemia remains a widespread public health problem in developed countries, it contributes significant proportion of children death in most developing countries including Ethiopia. As such, various factors like parasite have impact on cognitive development and physical growth, studies on the magnitude of anemia among under-five age children have paramount importance. Meanwhile, there is a limited study on the prevalence and associated factor of anemia among under-five age children in our study area. Therefore, this study aimed to determine the prevalence of anemia and its associated factors among under-five children in Shanan Gibe Hospital, Southwest Ethiopia.

## Methods

### Study area, design, and period

Institution based cross sectional study was conducted in Shanan Gibe Hospital , Jimma town, Ethiopia from 1 January to 30 April, 2021.

### Population

All under-five age children who attended in SGH were the source population. However, under five age children who visited SGH during the study period were study population and the under five children who provided stool sample and blood were study participant in the current study.

### Sample size and sampling technique

The sample size was calculated by using single population proportion formula by taking 41.1% prevalence of anemia among under five children in in Guguftu, South Wollo, Northeast Ethiopia [20] using the assumption of 95% confidence level (z = Za/2 = 95% = 1.96), margin of error (d = 5% = 0.05), Then, sample size was determined as follow:

$$\mathrm{N}=\frac{{\left({\mathrm{Z}}_{\infty /2}\right)}^2\mathrm{p}\ \left(1-\mathrm{p}\right)}{{\mathrm{d}}^2}=368$$

Therefore, the minimum of 368 study participants were selected by consecutive convenient sampling technique.

### Inclusion and exclusion criteria

All under five children attending to SGH and those provided stool sample were included to study. However, those under five children who did take drugs for deworming before one month of data collection were excluded to this study.

### Data collection and processing

English version questionnaire was translated in to Amharic version and finally it was translated back to English version to check its consistency. The data were collected by a trained nurse and a principal investigator. Thus, Socio demographic and possible associated risk factors of anemia were collected by structured pre-tested Amharic version questionnaire using face to face interview with parents/ guardians based on an interviewer-administered semi structured questionnaire. At each data collection spot, sufficient explanation about the aim of the research was given to the parents or study participants before conducting the interview.

### Sample collection and transportation

Whole blood and stool samples were collected aseptically. The whole blood was used for hemoglobin measurement immediately at site data collection in hospital, while the stool samples were transported from clinical services outlets to department of diagnostic laboratory in Shana Gibe Hospital (SGH).

### Hemoglobin measurement

The Hgb concentration of each participant was measured by taking a finger-prick blood sample using a portable hemoglobin meter instrument (calibrated HaemoCue digital photometer). Hemoglobin value was displayed within 15–20 s and interpreted as; Hgb < 11 g/dl were anemic and Hgb > 11 g/dl was defined as non-anemic for under five age children. The severity of anemia was categorized according to WHO cut-off value scheme as Hgb between 10.0 g/dl- 10.9 g/dl for mild anemia, Hgb between 7.0–9.9 g/dl for moderate anemia and Hgb less than 7 g/dl for severe anemia on under-five age children [21].

### Parasitic examination

After the interviewer administered questionnaire, the participants were sent to laboratory and asked them to bring about 10-*g* (teaspoonful amount) stool specimen was collected with wide mouth screw capped containers labeled with their identification code number for each study participant following the standard operating procedures (SOPs). The stool samples were examined for intestinal parasites using wet mount preparation on 10x objective microscopy within 10*–*15 *min* after collection. The remaining samples were stored in a cool box (regulated the temperature to <20 °C) and on the next day, formal-ether concentration technique was employed to diagnosis few parasitic density [22]. Finally, report and were record each parasite in the prepared laboratory format.

### Data analysis

Data was entered by EpiData version 3.1 and data analysis was performed using SPSS version 20. The prevalence of anemia was determined by descriptive statistics. Multivariable logistic regression was done by entering the variables with *p* < 0.2 in bivariable logistic regression to identify the factors associated with anemia. A *P* value <0.05 considered as statistically significant association in the multivariable logistic regression.

### Quality control

The data were collected by a trained nurse and principal investigators. Pre-test was done on 18 under five children at Jimma Medical Center before data collection and the actual study was performed to check acceptability of the questionnaire whether it contains the necessary information or not and if unnecessary, to make possible corrections..

Completeness of questionnaires was checked soon after collecting the data and calibrated HaemoCue digital photometer was used for Hgb concentration determination. The reagents were checked for their expired date before any test was performed. The patient’s blood and stool sample was collected, prepared and tested according to SOP to get reliable result. In each step, pre analytical, analytical and post analytical phases were maintained for quality assurance. At the end, the results were checked and registered on the laboratory record format before delivery to the patients.

### Ethical consideration

The study approved by College of Medicine and Health Science, Jimma University’s Research Institutional Review Board with reference number RU 1986/07 and a permission letter was obtained from Shanan Gibe Hospital. The purpose and importance of the study was explained to the participants. Written informed consent from parent/guardian was obtained in accordance with the Declaration of Helsinki. Additionally, absence of link between the study and their service was explained and participation was entirely voluntary based. Furthermore, the confidentiality of study participant was kept and identification of study participant by name was avoided. Finally, all participants who were diagnosed positive for intestinal parasites and anemia were linked to Medical doctors in Hospital for further management.

### Operational definitions

**Under five children:** the children whose ages are less than five years.

**Hemoglobin** – A red substance in the blood that carries oxygen and contains iron.

**Non anemic** - Hemoglobin concentrations greater than 11.0 g/dl in the blood of the individual.

**Anemic** - Children with Hgb level < 11.0 g/dL.

**Mild anemia** – Hemoglobin concentrations in between 10–10.9 g/dl in the blood of the individual.

**Moderate anemia** – Hemoglobin concentrations in between 7.0 g /dl-9.9 g/dl in the blood of the individuals.

**Severe anemia**– is hemoglobin concentrations which is less than 7.0 g /dl in the individual’s blood.

**Monthly income:** it was categorized based Ethiopian civil service proclamation as individual earning with low income (<3000 ETB), medium income (3000–7500 ETB) and high income (>7500 ETB).

## Results

### Socio-demographic characteristics of study participants

A total of 368 under-five age children were participated in the study. Of these, about 195 (52.9.0%) were females and 147 (39.9%) were within the age group of 2**–**3 years. According to educational status of their mothers, the highest (47.0%) of the children’s mothers were unable to read and write. However, the family monthly income with 247(67.1%) had low income and 1**–**5 family size accounted 228 (62.0%). The other socio demographic characteristics were summarized below (Table 1).

**Table 1:** Socio demographic distribution of under-five age children visited in Shanan Gibe Hospital, Southwestern Ethiopia, 1 January to 30 April, 2021

Characteristics	Category	Frequency	Percent (%)
Sex	Male	173	47.0
	Female	195	53.0
Age	0–1	87	23.6
	2–3	147	40.0
	4–5	134	36.4
Educational status (Mothers)	Unable to read and write	173	47.0
	Primary (1–8)	9	26.4
	Secondary (9–12)	28	7.6
	>Grade 12	70	19.0
Residence (mother)	Urban	212	57.6.0
	Rural	156	42.4
Monthly income (mother)	Low income (<3000 ETB)	247	67.1
	Medium income (3000–7500 ETB)	76	20.7
	High income (>7500 ETB)	45	12.2
Family size	1–5	228	62.0
	>5	140	38.0

*Note*: *ETB* Ethiopian Birr

### Prevalence of anemia

**Fig. 1:**
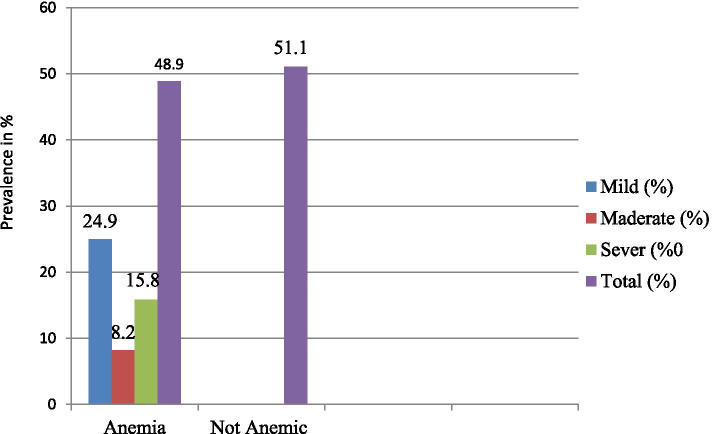
Grade and prevalence of anemia among under five children in Shanan Gibe Hospital, Southwestern Ethiopia, January 1 January to 30 April, 2021

The prevalence of anemia in this study was 180 (48.9%) with [95% CI: 39.24–53.19]. The degree of Anemia was classified as mild, moderate, & severe anemia according to WHO cutoff values for grading anemia (43). Thus, from the total of 180 (48.9%) anemic children, 92 (24.9%) and 58 (15.8%) and 30 (8.2%) were mild, moderate and severe anemia, respectively (Fig. 1).

### Proportion of intestine parasites

**Fig. 2:**
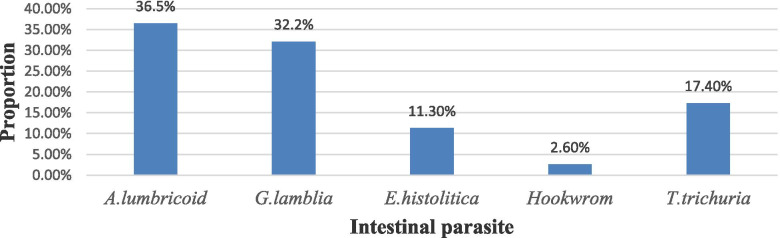
Magnitude of intestinal parasite among under five age children visited in Shanan Gibe Hospital, Southwestern Ethiopia, 1 January to 30 April, 2021

The proportions of intestinal parasite infection was 115 (31.25%) with (95%CI = 30.28–72.42). From these, the proporation of each intestinal parasites were recorded as *Ascaris lumbriciods* (*A. lumbricoide*) 42 (36.5%), *Gardia lamblia* (*G. lamblia*) 37 (32.2%), *Enteameba histolytica* (*E. histolytica*) 13(11.3%), Hookworm 3 (2.60%) and *Trichuris trichuria* (*T. trichuria*) 20 (17.40%) as shown below (Fig. 2).

### Factor associated with anemia

All independent variables showing *P*-value <0.2 in the bivariable analysis including age, residence, mother monthly income, family size, *A. lumbricoide, G. lamblia, E. histolytica,* Hookworm *and T.trichuria* were entered in to multivariable logistic regression analysis to determine factors associated with anemia among under-five age children. Accordingly, only being rural residence (AOR = 6.11; 95% CI = 1.49–8.99, *P* = 0.002), family low income (AOR = 6.27, 95% CI = 1.35–11.43, *P* = 0.04) and family size greater than five (AOR = 3.12; 95% CI =1.47–7.11, *P* = 0.002) *P* < 0.05 were demonstrated as significant association with anaemia among under five children. Thus, the adjusted odds of children with rural residence having anemia was 6.11 times higher compared to those from urban residents.. Similarly, the adjusted odds of children with low income mothers having anemia was 6.27 times higher compared to those children from mothers having high monthly income. Additionally,, the adjusted odds of children with in from greater than five family size in home having anemia was 3.12 times higher compared to those children form family size less than five in home (Table 2).

**Table 2:** Factors associated with anemia among under five age children in Shanan Gibe Hospital, Southwestern Ethiopia, 1 January to 30 April, 2021

Variables	Category	Anemic (n = 180)	Non-anemic (n = 188)	COR(95%CI) *P* value	AOR(95%CI)	*P* value
		N (%)	N (%)			
Sex	Male	82 (47.4)	91 (52.6)	1	–	–
	Female	98 (50.3)	97 (49.7)	3.21 (0.67–8.12)0.88		
Age	0–1	37 (42.5)	50 (57.5)	7.12 (1.93–11.32)0.001*	5.31 (0.9–10.13)	0.17
	2–3	77 (52.4)	70 (47.6)	3.27 (2.5–8.45)0.08*	4.62 (0.85–7.44)	0.44
	4–5	66 (49.3)	68 (50.7)	1	1	
Educational statues (mother)	Unable read & write	93 (53.8)	80 (46.2)	4.12 (0.69–12.71)0.91	–	–
	primary (1–8)	41 (42.3)	56 (57.7)	5.15 (0.72–6.88)0.53		
	Secondary (9–12)	10 (35.7)	18 (64.3)	2.32 (0.93–5.78)0.31		
	>Grade 12	36 (51.4)	34 (48.6)	1		
Residence	Urban	114 (53.8)	98 (46.2)	3.57 (1.09–7.55)0.001*	6.11 (1.49–8.99)	0.002**
	Rural	66 (42.3)	90 (57.7)	1	1	
Monthly income (mother)	Low income	128 (51.8)	119 (48.2)	7.34 (1.77–11.9)0.06*	6.27 (1.35–11.43)	0.004**
	Medium income	28 (36.8)	48 (63.2)	4.51 (1.21–8.02)0.014*	2.17 (1.83–8.05)	0.41
	High income	24 (53.3)	21 (46.7)	1	1	
Family size	1–5	116 (50.9)	112 (49.1)	1	1	
	>5	64 (45.7)	76 (54.3)	4.55 (1.33–6.71)0.001*	3.12 (1.47–7.11)	0.002**
Utilize latrine	Yes	60 (51.3)	57 (48.7)	0.78 (0.42–5.38)0.58	–	–
	No	120 (47.8)	131 (52.2)	1		
Hand wash before meal	Yes	69 (60.0)	46 (40.0)	2.93 (0.39–7.09)0.73	–	–
	No	111 (43.9)	142 (56.1)	1		
Finger trimming	Yes	72 (54.1))	61 (45.9)	3.23 (0.99–8.02)0.44	–	–
	No	108 (46.0)	127 (54.0)	1		
*A. lumbricoide*	Yes	42 (91.3)	4 (8.7)	4.51 (2.13–7.54)0.006*	3.76 (0.82–5.98)	0.21
	No	138 (42.9)	184 (57.1)	1	1	
*G. lamblia*	Yes	37 (92.5)	3 (7.5)	1.35 (1.21–7.25)0.01*	1.45 (0.9–8.12)	0.57
	No	143 (43.6)	185 (56.4)	1	1	
*E. histolytica*	Yes	13 (86.7)	2 (13.3)	2.57 (2.1–9.13)0.001*	3.37 (2.16–11.31)	0.005**
	No	167 (47.3)	186 (52.7)	1	1	
Hookworm	Yes	3 (75.0)	1 (25.0)	4.75 (3.21–10.92)0.001*	6.09 (2.37–11.56)	0.001**
	No	177 (48.6)	187 (51.4)	1	1	
*T.trichuria*	Yes	20 (74.1)	7 (25.9)	3.53 (2.67–8.23)0.001*	2.79 (1.45–9.13)	0.002**
	No	160 (46.9)	181 (53.1)	1	1	

Key: *AOR* Adjusted Odd ratio, *COR* Crude Odd Ratio, *N* Frequency, % Percent, *CI* Confidence interval, *1* reference,* = variables selected for multivariable logistic regression, ** = significantly associated variable

On the other hand, some intestinal parasites were significantly associated with anemia. Hence, *E. histolytica* (AOR =3.37; 95%CI = 2.16**–**11.31, *P* = 0.005), Hookworm (AOR = 6.09; 95%CI = 2.37**–**11.56, *P* = 0.001),and *T. trichuria* (AOR = 2.79; 95%CI = 1.45**–**9.13, *P* = 0.002) were the indentified intestinal parasites associated with anemia among under five children by considering *P* value less than 0.05 as statistical significantly association (Table 2).

## Discussion

Anemia has been continued to be a health problem on under five children [23] which adversely affects mental, physical and social development of the children [20]. This burden also affected middle and low income countries including Ethiopia in particular our study area. Hence, the prevalence of anemia was 48.9% at 95% CI (39.24–53.19) in the current study. As the result, other previous studies were compared with our study by categorizing in to low, comparable and high prevalence on the bases of CI of this study.

Accordingly, the prevalence of anemia in the current study was higher than previous studies reported in Huaihua (29.73%) [24], Senegal (30.7%) [25], Uganda 26·2% [15], Tanzania at Rombo district 37.9% [26]. The variation might be due to the variability of risk factors across different geographic regions, plus lower socioeconomic, large family size and maternal education status of under-five age children those factor which contribute increase the prevalence of anemia.

The current anemia prevalence among under five children was analogous with studies indicated in Palestine 33.5% [27], South Kivu 39.6% [18], Ghana (41%) [28], Cape Verde 51.8% [29] and in Ethiopia (44.83%) [30], Wollo (41.1%) [20], Filtu Town, Somali region (41.7%) [31], Duggina Fanigo District of Wolaita Zone 51.4% [32] and Amhara region (41.43%) [33]. In contrast, a high prevalence e of anemia had been reported in sub-Saharan Africa (64.1%) [34], East Africa (75%) [35], Brazil (56.6%) [36], Malawi 56.9% [4], Togo (70.9%) [37], Tanzania, Bugando Medical Centre (77.2%) [38], Arusha District in Tanzania (84.6%) [39], Kenyan Coast (76·3%) [40], Mali (58%), Tanzania (57%), and Mozambique (54%) [27], Ghana 78.4% [41]. This variation might be due to the mothers having the problem with their low monthly income or parasitic infestations of children which are contributing factors associated with lower hemoglobin (Hgb) level in children.

According to factors in the current study, being rural residence (AOR = 6.11; *P* = 0.002) was associated with anemia in the current study. Beside this, analogs association of rural residence finding was reported in Amhara region [33, 42]. As such, studies indicated that the distribution of anemia is more prevalent among children from rural resident compared to urban ones [43]. This is because of low socioeconomic status, low serving of iron-rich foods, lack of adequate nutrition information or dietary intake and due to and a high number of illiterates in rural areas as compared to urban [44].

Family low income (AOR = 6.27; *P* = 0.004) was the identified associated factor with anemia among under five children in current study which was previously reported in Ethiopia like Wollo [20] and Filtu Town, Somali region [31]. This factor could be associated with anemia due to high family income with higher wealth quintile are more likely to provide balanced macro and micronutrients (minerals and vitamins) to their children and children from a lower economic status are vulnerable to various nutritional disorders including anemia [45]. On the other hand, family size greater than five (AOR = 3.12; *P* = 0.002) *P* < 0.05 was another identified factors associated with anemia among under five children which was founded in sub-Saharan Africa [34]. So, having large family size was reported factor associated with as anemia due to an increase in the number of children might lead to a risk of communicable disease transmission, and competition for food, consequently, nutritional deficiencies [46] Thus, children with rural residence, low income family and family size greater than five in home were 6.11, 6.27 and 3.12 times more likely to develop anaemia among under five children by adjusting other confounding factors, respectively.

On the other hand, intestinal parasites such as *E. histolytica* (AOR =3.37; *P* = 0.005), Hookworm (AOR = 6.09; *P* = 0.001), and *T. trichuria* (AOR = 2.79; *P* = 0.002) contributed to anemia in children. Similar findings of *E. histolytica* was report in Health Center, North Ethiopia [47], while Hookworm findings also reported in Kenyan coast [39], Ethiopia particularly, Yirgacheffee [48] and Harbu Town [49]. This association might be due to significantly low level of serum iron in *E. histolytica* infected children due to *E. histolytica* needs great levels of iron to stay alive and reproduction [50]. *T. trichiura* and hookworm can cause massive amounts of blood loss and decreased hemoglobin levels [51]. So, anemia is positively associated with intestinal parasite which might be due to lack of hygiene related practices, Red Blood Cell (RBC) destruction and feeding, and loss of appetite caused by worms [52]. Similarly, the blood loss that can occur in *T. trichiura* infection is likely to contribute to anemia [51, 53]**.**

As the result, children infected with *E. histolytica,* Hookworm and *T. trichuria* were 3.37, 6.09 and 2.79 times more likely to develop anaemia by adjusting other confounding factors, respectively**.** This study is limited due to the fact that present study didn’t differentiate the types of anemia as it was due to iron, vitamin B12, folic deficiency or the RBC morphological effect and hemoparasites were not assessed for factors associated with anemia among under five children.

## Conclusion

The prevalence of anemia among under five age children was high. The study also revealed that the most important associated factors for anemia are rural resident, family size greater than five in home, low family income, infection with *E. hislotytica*, Hookworm and *T. trichuria* are the factors associated with anemia in this study. Therefore, there should be a continuous health education for their parents on factors of anemia in addition to massive and routine deworming for intestinal parasites to reduce the burden of anemia among children in hospitals.

## Data Availability

Results of this study are generated from the data collected and analyzed based on stated materials and methods. All data concerning to this study could be available upon the request of Destaw Kebede (Correspondence author; mobile +251911594675, email: amaueldestaw@gmail.com).

## Abbreviations

DHS: Demographic and heath survey
ETB: Ethiopian Birr
Hgb: Hemoglobin
IP: Intestinal Parasite
SGH: Shanan Gibe Hospital
SOP: Standard Operational Procedure
SPSS: Statistical Program for Social Science
STH: Soil Transmitted Helminthes
WHO: World Health Organization
